# Tripole-mode and quadrupole-mode solitons in (1 + 1)-dimensional nonlinear media with a spatial exponential-decay nonlocality

**DOI:** 10.1038/s41598-017-00197-6

**Published:** 2017-03-09

**Authors:** Zhiping Dai, Zhenjun Yang, Xiaohui Ling, Shumin Zhang, Zhaoguang Pang, Xingliang Li, Youwen Wang

**Affiliations:** 1 College of Physics and Electronic Engineering, Hengyang Normal University, Hengyang, 421002 China; 2 College of Physics and Information Engineering, Hebei Advanced Thin Films Laboratory, Hebei Normal University, Shijiazhuang, 050024 China

## Abstract

The approximate analytical expressions of tripole-mode and quadrupole-mode solitons in (1 + 1)-dimensional nematic liquid crystals are obtained by applying the variational approach. It is found that the soliton powers for the two types of solitons are not equal with the same parameters, which is much different from their counterparts in the Snyder-Mitchell model (an ideal and typical strongly nolocal nonlinear model). The numerical simulations show that for the strongly nonlocal case, by expanding the response function to the second order, the approximate soliton solutions are in good agreement with the numerical results. Furthermore, by expanding the respond function to the higher orders, the accuracy and the validity range of the approximate soliton solutions increase. If the response function is expanded to the tenth order, the approximate solutions are still valid for the general nonlocal case.

## Introduction

The nonlocal nonlinearity exsits naturally in many physical systems, such as nematic liquid crystals (NLCs)^1–4^, lead glasses^5^, atomic vapors^6^, Bose-Einstein condensates^7^, and photorefractive crystals^8^. In the past years, solitons in nonlocal nonlinear media have attracted much attention. In nonlocal nonlinear media, the beam propagation is governed by the nonlocal nonlinear Schrödinger equation (NNLSE)^1, 4, 9–11^. For the strongly nonlocal case, Snyder and Mitchell reduced the NNLSE to a linear model, i.e., Snyder-Mitchell model (SMM)^12^. Subsequently, many solitons are found theoretically in SMM^12–16^. However, the SMM is an ideal model and it is still not realized in experiments. The inadequacies of the SMM have been discussed for the actual experimental regime for nonlinear optical beams in nematic liquid crystals^10^. It has been found that the NLCs is a typical nonlocal nonlinear medium which actually exists, and its degree of nonlocality is controllable by changing the pretilt angle of NLCs via a bias voltage^1, 3, 17^. Hence many experiments are carried out in NLCs, including the first observation of nonlocal solitons^9^. The solitons in NLCs are also called nematicons which was invented by Prof. G. Assanto in 2003 and used thereafter^4, 18^. In NLCs, there exist various novel solitons, for instance dark nematicons^19^, nematicon-vortex vector solitons^20^, multipole solitons^21, 22^, which exhibit many unique characteristics, such as the effect of deflection^20, 23^ and the novel all-optical switching based on interacting nematicons^24–26^, etc. Especially, two solitons in strongly nonlocal nonlinear media always attract each other whatever their relative phase is refs 27–30, even for two out-phase bright/dark solitons^3, 31^, which is distinctly different from the case in local nonlinear media, where two out-of-phase solitons repel each other.

The nonlocal response in (1 + 1)-dimensional NLCs can be described by an exponential-decay function^31^, while in (2 + 1)-dimensional NLCs, it is expressed as the zeroth order modified Bessel function^3^. Although some exact solutions can be obtained by solving nonlinear equations directly, it is indeed a difficult task. Variational approach is a valid method to approximately solve some nonlinear equations, which was first introduced into optics by Anderson^32^. Minzoni *et al*. first used the variational approach to find an approximate solution for a solitary wave in a nematic liquid crystal^33^, based on the previous work for the NLS equation performed by Kath *et al*.^34^. The work in refs 33 and 34 does more than find the steady solitary wave, it also finds the evolution to this solitary wave from an initial condition^33, 34^. Malomed presented a general review of these variational methods in nonlinear fiber optics and related fields^35^. Recently, Aleksić *et al*. analytically investigated the fundamental solitons based on the variational approach in (2 + 1)-dimensional NLCs^36^. Especially, MacNeil *et al*. obtained exact solutions of the nematicon equations in (1 + 1) and (2 + 1) dimensions for fixed parameter values, and they also got approximate solutions based on the variational approach method^37^. Furthermore, Panayotaros and Marchant addressed the existence of a solitary wave solution of the nematic equations mathematically^38^. It has been proven that in the media with an exponential-decay nonlocal response, the soliton bound states are stable if the solitons contain fewer than five-poles^21^. Namely the fundamental, dipole-mode, tripole-mode and quadrupole-mode solitons can all propagate stably in such media. The fundamental and dipole-mode solitons in nonlinear media with an exponential-decay nonlocal response have been investigated analytically by various mathematical methods, such as the classical Lie-group method^39^, the perturbative analysis method^40^, and the variational approach method^36, 37, 41, 42^. But so far, no one gives analytical expressions of tripole-mode and quadrupole-mode solitons in NLCs. Presenting an analytical solution is helpful for one to get a good understanding of the dynamics of nonlocal solitons. In this paper, based on the variational approach, we study the tripole-mode and quadrupole-mode solitons in nonlinear media with an exponential-decay nonlocal response. The approximate expressions of such solitons are obtained and the characteristics of them are investigated in detail. The approximate results are confirmed by the numerical ones which are obtained using the iterative numerical technique based on the NNLSE directly. Since a surface soliton in nonlocal nonlinear media can be regarded as a half of a bulk soliton with an antisymmetric amplitude distribution^43, 44^, the results on quadrupole-mode solitons here may also be helpful for the investigation of the surface dipole nonlocal solitons.

## Results

### Variational method for NNLSE and tripole-mode soliton solutions

First of all, let us roughly recall the derivation of the dimensionless NNLSE for the (1 + 1)-dimensional NLCs based on refs 1, 31 and 45. Considering only one transversal dimension^45^, an external quasistatic electric field *E*_*LF*_ is applied in the transversal direction to control the initial tilt angle of the NLCs. The evolution of an optical beam *Q*(*X*, *Z*) in the paraxial approximation and optically induced reorientation angle perturbation ${\rm{\Psi }}(X,Z)$ of the liquid crystal molecules can be described as follows^1, 31^

1 $$ik\frac{\partial Q}{\partial Z}+\frac{1}{2}\frac{{\partial }^{2}Q}{\partial {X}^{2}}+\frac{{k}_{0}^{2}}{2{\varepsilon }_{0}}{\rm{\Delta }}{\varepsilon }_{HF}{\rm{\Psi }}Q=0,$$

2 $$K\frac{{\partial }^{2}{\rm{\Psi }}}{\partial {Z}^{2}}+K\frac{{\partial }^{2}{\rm{\Psi }}}{\partial {X}^{2}}-2\frac{{\rm{\Delta }}{\varepsilon }_{LF}{E}_{LF}^{2}}{\pi }{\rm{\Psi }}+\frac{{\rm{\Delta }}{\varepsilon }_{HF}}{4}{|Q|}^{2}=0,$$

where *k*_0_ and *k* are, respectively, the wave numbers in vacuum and the NLCs. Δ*ε*_*HF*_ and Δ*ε*_*LF*_ are, respectively, the anisotropy of the liquid crystal at the optical frequency and the anisotropy of the liquid crystal at the frequency of the quasistatic electric field. *ε*_0_ is the vacuum permittivity. *K* is the relevant elastic constant taken equal for splay, bend, and twist.

Introducing the normalization: *x* = *X*/*W*_0_, *z* = *Z*/*Z*_*R*_, *q* = *Q*/*Q*_0_, and $\psi ={\rm{\Psi }}/{{\rm{\Psi }}}_{0}$, where *W*_0_ is the full width at half maximum of the amplitude of the optical beam, ${Z}_{R}=k{W}_{0}^{2}$ is the Rayleigh distance, ${Q}_{0}=2{E}_{LF}\sqrt{2{\varepsilon }_{0}{\rm{\Delta }}{\varepsilon }_{LF}/\pi }/({\rm{\Delta }}{\varepsilon }_{HF}{k}_{0}{W}_{0})$, and ${{\rm{\Psi }}}_{0}={\varepsilon }_{0}/({k}_{0}^{2}{W}_{0}^{2}{\rm{\Delta }}{\varepsilon }_{LF})$, one can obtain the following dimensionless equations

3 $$i\frac{\partial q}{\partial z}+\frac{1}{2}\frac{{\partial }^{2}q}{\partial {x}^{2}}+\psi q=0,$$

4 $${\sigma }^{2}\frac{{\partial }^{2}\psi }{\partial {x}^{2}}-\psi +{|q|}^{2}=0,$$

where *σ* denotes the degree of nonlocality which is expressed as^31^

5 $${\sigma }^{2}=\frac{\pi K}{2{W}_{0}^{2}{E}_{LF}^{2}{\rm{\Delta }}{\varepsilon }_{LF}}.$$

In Eq. (4), the term ∂^2^ *ψ*/∂*z* ^2^ has been canceled since ${W}_{0}/{Z}_{R}\ll 1$, which is a part of the coefficient multiplying the derivative *ψ*_*zz*_ in the non-dimensional director equation^1, 3^. Based on Eq. (4), combined with Fourier transformation and the convolution theorem, one can find

6 $$\psi (x,z)=\frac{1}{2}{\int }_{-\infty }^{+\infty }R(x-x^{\prime} )I(x^{\prime} )dx^{\prime} ,$$

where *I* = |*q*|^2^ is the intensity of the optical beam, and the normalized nonlocal response function *R* takes an exponential-decay function as follows

7 $$R(x)=\frac{1}{2\sigma }\exp (-\frac{|x|}{\sigma }).$$

Several kinds of solitons in (1 + 1)-dimensional nonlinear media with a spatial exponential-decay nonlocality has been investigated in the past years^46, 47^. In experiments^4, 9–11^, the typical values of the parameters are: *W*_0_ = 10 *μ*m, *K* = 10^−11^ N, Δ*ε*_*HF*_ = 0.64*ε*_0_, Δ*ε*_*LF*_ = 15*ε*_0_, *E*_*LF*_ = 10^4^ V/m. For the visible wavelengths, *ε*_0_ is 8.85 × 10^−12^ in MKS units, and the diffraction length is about 1 mm. With the above parameters, the degree of nonlocality is about $\sqrt{12}$, i.e. ${\sigma }^{2}\approx 12$, which belongs to the sub-strongly nonlocal case.

If *σ* → *w*_*m*_ and *ψ* → Δ*n*, the dimensionless NNLSE, which governs the beam propagation in (1 + 1)-dimensional nonlinear media with an exponential-decay nonlocal response, can be rewritten phenomenologically as follows^17, 31^

8 $$i\frac{\partial q}{\partial z}+\frac{1}{2}\frac{{\partial }^{2}q}{\partial {x}^{2}}+{\rm{\Delta }}nq=\mathrm{0,}$$

9 $${\rm{\Delta }}n-{w}_{m}^{2}\frac{{\partial }^{2}{\rm{\Delta }}n}{\partial {x}^{2}}={|q|}^{2},$$

where *x* and *z* denote, respectively, the normalized transversal and longitudinal coordinates; *q* denotes the complex amplitude of the optical beam; Δ*n* denotes the nonlinear perturbation of the refractive index of the nonlocal medium; *w*_*m*_ denotes the characteristic length of the nonlocal material response. If *w*_*m*_ → 0, Eq. (8) is simplified to the well-known nonlinear Schrödinger equation in local nonlinear media; if *w*_*m*_~*w*_*R*_ (where *w*_*R*_ is the second-order moment width of multipole solitons), it represents the general nonlocal case; and if *w*_*m*_ → ∞, it represents the strongly nonlocal case^48, 49^. In experiments, the magnitude of *w*_*m*_ can be controlled by changing the pretilt angle of NLCs via a bias voltage^1, 3, 17^. Based on the above derivation, Eq. (8), together with Eq. (9), denotes a NNLSE with an exponential-decay nonlocal response^31^, which can be restated as follows,

10 $$i\frac{\partial q}{\partial z}+\frac{1}{2}\frac{{\partial }^{2}q}{\partial {x}^{2}}+q{\int }_{-\infty }^{+\infty }R(x-x^{\prime} ){|q(x^{\prime} ,z)|}^{2}dx^{\prime} \mathrm{=0,}$$

where

11 $$R(x)=\frac{1}{2{w}_{m}}\exp (-\frac{|x|}{{w}_{m}})$$

is the material response function. Equation (10) conserves energy flow $P={\int }_{-\infty }^{+\infty }{|q|}^{2}dx$ (i.e. the soliton power).

Equation (10) can be regarded as an Euler-Lagrange equation which corresponds to the variational problem

12 $$\delta {\int }_{0}^{+\infty }{\int }_{-\infty }^{+\infty } {\mathcal L} (q,{q}^{\ast },\frac{\partial q}{\partial x},\frac{\partial {q}^{\ast }}{\partial x},\frac{\partial q}{\partial z},\frac{\partial {q}^{\ast }}{\partial z})dxdz=0,$$

where the Lagrangian density can be expressed as

13 $$ {\mathcal L} =\frac{i}{2}({q}^{\ast }\frac{\partial q}{\partial z}-q\frac{\partial {q}^{\ast }}{\partial z})-\frac{1}{2}{|\frac{\partial q}{\partial x}|}^{2}+\frac{1}{2}{|q|}^{2}{\int }_{-\infty }^{+\infty }R(x-x^{\prime} ){|q(x^{\prime} )|}^{2}dx^{\prime} .$$

Substituting Eq. (13) into Eq. (12), the reduced variational problem can be obtained as follows

14 $$\delta {\int }_{0}^{+\infty }[ {\mathcal L} ]dz=0,$$

where the average Lagrange is expressed as

15 $$[ {\mathcal L} ]={\int }_{-\infty }^{+\infty } {\mathcal L} dx.$$

For the strongly nonlocal media (especially for the Snyder-Mitchell model), the higher-order Gaussian solitons (such as Hermite-Gaussian solitons, Laguerre-Gaussian solitons, Ince-Gausian solitons etc.) are the exact soliton solutions^12, 15, 50, 51^. For (1 + 1)-dimensional nonlinear media with a spatial exponential-decay nonlocality, we consider that solitons come into being from the Hermite-Gaussian beams. In our previous research, we have proven that the first-order Hermite-Gaussian function can be used to describe the dipole solitons in such nonlocal media^52^. Hence, we take the second- and third-order Hermite-Gaussian functions as the trial functions of tripole and quadrupole solitons, respectively. The trial solution of tripole-mode solitons takes the following form

16 $$q(x,z)=a(\frac{2{x}^{2}}{{w}^{2}}-1)\exp (-\frac{{x}^{2}}{2{w}^{2}}){e}^{i\theta (z)},$$

where *a* is the amplitude, *θ*(*z*) is the phase, and *w* is the width of a Gaussian soliton. Because of the complexity of the intensity distribution, the second-order moment beam width is adopted to describe the width of multipole solitons. The second-order moment beam widths is defined as

17 $${w}_{R}(z)={[\frac{4}{P}{\int }_{-\infty }^{+\infty }{x}^{2}{|q(x,z)|}^{2}dx]}^{\frac{1}{2}}.$$

Thus the width of a tripole-mode soliton is ${w}_{R}=\sqrt{10}w$. For the sake of convenience in the following discussion, we introduce a nonlocal parameter *α* to define the degree of the material nonlocality, i.e., *α* = *w*_*m*_/*w*_*R*_. The larger the nonlocal parameter, the stronger the degree of nonlocality.

In theory, based on Eqs (11), (13), (15) and (16), one can obtain the expression of $[ {\mathcal L} ]$. However, the integrals in the averaged Lagrangian based on the trial function could not be calculated explicitly due to the inability to find closed form integrals. Fortunately, for the strongly nonlocal case, we can calculate it by expanding the response function. If it is expanded to the second order, one can get

18 $$R(x)\simeq \frac{1}{2{w}_{m}}(1-\frac{|x|}{{w}_{m}}+\frac{{x}^{2}}{2{w}_{m}^{2}}).$$

Substituting Eqs (13), (16) and (18) into Eq. (15), the expression of $[ {\mathcal L} ]$ is obtained as follows

19 $$[ {\mathcal L} ]=-\frac{5\sqrt{\pi }{a}^{2}}{2w}+\frac{{a}^{4}{w}^{2}(160\pi {w}^{2}-145\sqrt{2\pi }w{w}_{m}+64\pi {w}_{m}^{2})}{64{w}_{m}^{3}}-2{a}^{2}\sqrt{\pi }w\theta ^{\prime} (z),$$

where $\theta ^{\prime} (z)={\theta }_{z}=\partial \theta (z)/\partial z$. Based on the corresponding Euler-Lagrangian equations, one can get

20 $$-\frac{5\sqrt{\pi }a}{w}+\frac{{a}^{3}{w}^{2}[-145\sqrt{2\pi }w{w}_{m}+32\pi (5{w}^{2}+2{w}_{m}^{2})]}{16{w}_{m}^{3}}-4a\sqrt{\pi }w\theta ^{\prime} (z)=0,$$

21 $$160{w}_{m}^{3}+{a}^{2}{w}^{3}[-435\sqrt{2}w{w}_{m}+128\sqrt{\pi }(5{w}^{2}+{w}_{m}^{2})]-128{w}^{2}{w}_{m}^{3}\theta ^{\prime} (z)=\mathrm{0,}$$

As we know, for the soliton case, *θ*′(*z*) = *βz*, where *β* is the propagation constant. Combining Eqs (20) and (21), one can obtain

22 $$a=\frac{4\sqrt[4]{8}\sqrt{{w}^{3}}}{\sqrt{-32\sqrt{2\pi }{w}^{5}+29{w}^{4}{w}_{m}}},$$

23 $$\beta =\frac{1}{4}(-\frac{15}{{w}^{2}}-\frac{4{w}_{m}^{2}}{{w}^{4}}-\frac{290{w}^{2}{w}_{m}-116{w}_{m}^{3}}{-32\sqrt{2\pi }{w}^{5}+29{w}^{4}{w}_{m}}),$$

and the soliton power

24 $$P=\frac{64\sqrt{2\pi }{w}_{m}^{3}}{(29{w}_{m}-32\sqrt{2\pi }w){w}^{3}}.$$

It is evident that Eqs (22–24) are valid only when $32\sqrt{2\pi }{w}^{5} < 29{w}^{4}{w}_{m}$. If $32\sqrt{2\pi }{w}^{5} > 29{w}^{4}{w}_{m}$, *a* becomes an imaginary number, *β* < 0, and *P* < 0, which is impossible in physics.

**Figure 1:**
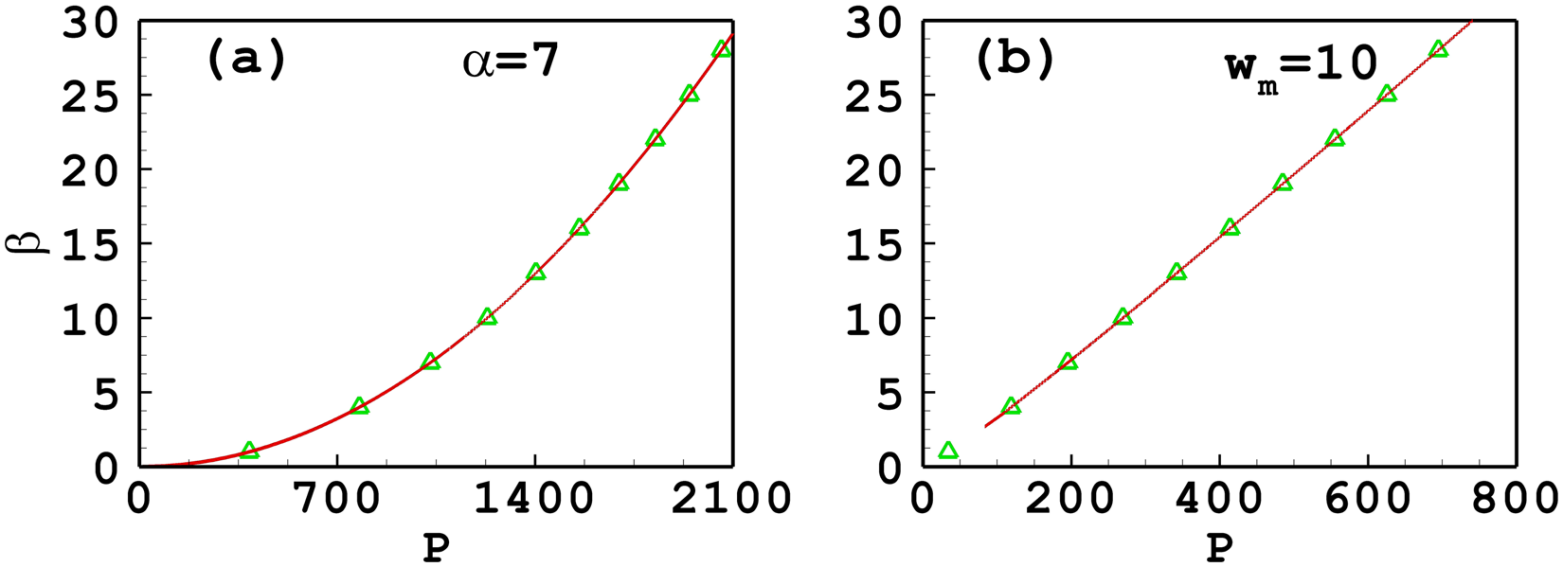
The propagation constant versus the power of tripole-mode solitons. (**a**) The degree of nonlocality is fixed at *α* = 7; (**b**) the width of the response function is fixed at *w*_*m*_ = 10. The solid line and the triangles denote, respectively, the approximate solutions and the numerical ones.

**Figure 2:**
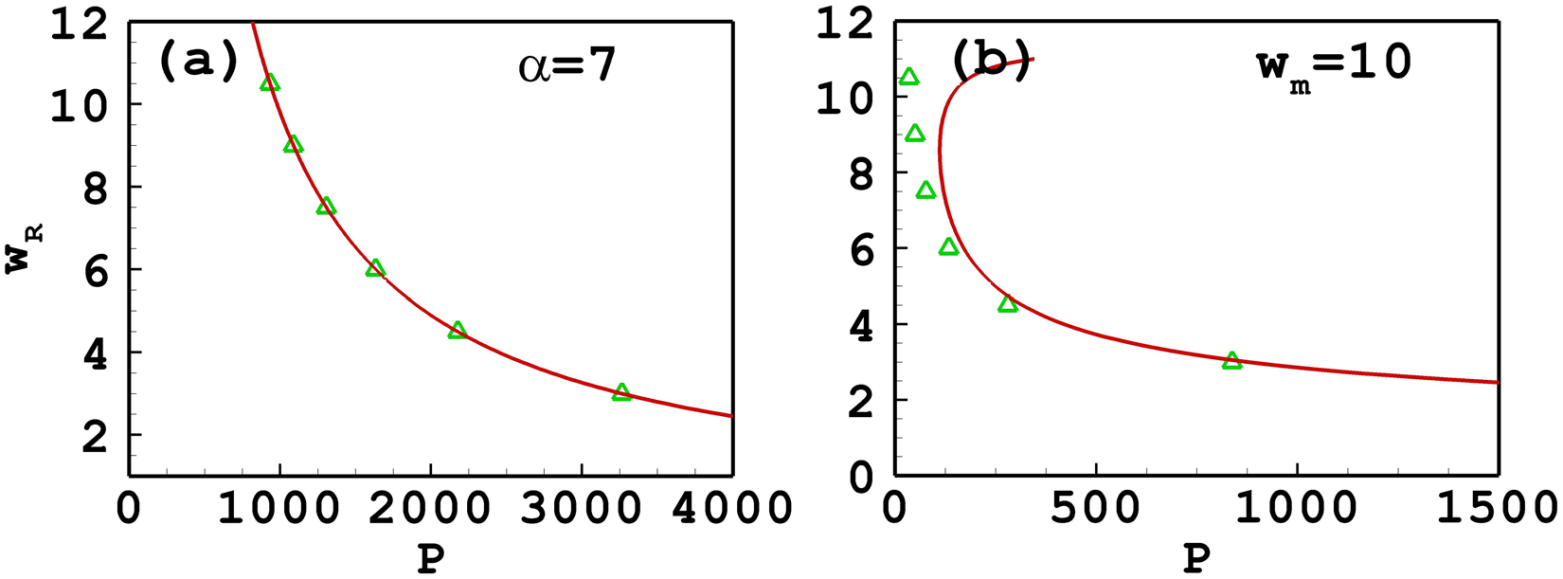
The soliton width versus the power of tripole-mode solitons. (**a**) The degree of nonlocality is fixed at *α* = 7; (**b**) the width of the response function is fixed at *w*_*m*_ = 10. The solid line and the triangles denote, respectively, the approximate solutions and the numerical ones.

Figure 1 shows the propagation constant of tripole-mode solitons versus the soliton powers. In Fig. 1(a), the degree of nonlocality is 7, which belongs to the strongly or at least sub-strongly nonlocal case. It is found that the approximate result is in good agreement with the numerical one which is obtained directly based on Eqs (8) and (9) using the iterative numerical technique^53^. When *w*_*m*_ is fixed at 10, the approximate result is also in good agreement with the numerical ones as shown in Fig. 1(b). Figure 1(b) also shows that the approximate result it is invalid when *β* < 3.68 as the variational solution (22)–(24) breaks down, as discussed after Eq. (24). The reason for the invalidity is that the response function is only expanded to second order [see Eq. (18)], which leads to the inaccuracy. By expanding the respond function to higher orders, one can improve the accuracy of the approximate solutions, which will be discussed in the following Section. In addition, one can find from Fig. 1 that the slope of the power versus propagation constant is positive, which implies that the soliton propagation is stable. It is also found that if *w*_*m*_ takes a larger value, the valid region of *β* also becomes larger. The accuracy of approximate results is only dependent on the degree of nonlocality. The stronger the degree of nonlocality, the more accurate the approximate results. As a result, when the degree of nonlocality is still fixed at 7, the analytical result is always accurate independent of the soliton width [see Fig. 2(a)]. Nevertheless, if *w*_*m*_ is fixed, the degree of nonlocality decreases with the increase of the soliton width, so the validity of approximate results is getting declined continuously [see Fig. 2(b)]. The variational approximation shows bistability in Fig. 2(b), while the numerical solution does not. This is an important point as it shows that the variational approximation can predict behaviour which is not actually present. Caution should then be exercised with variational approximations.

**Figure 3:**
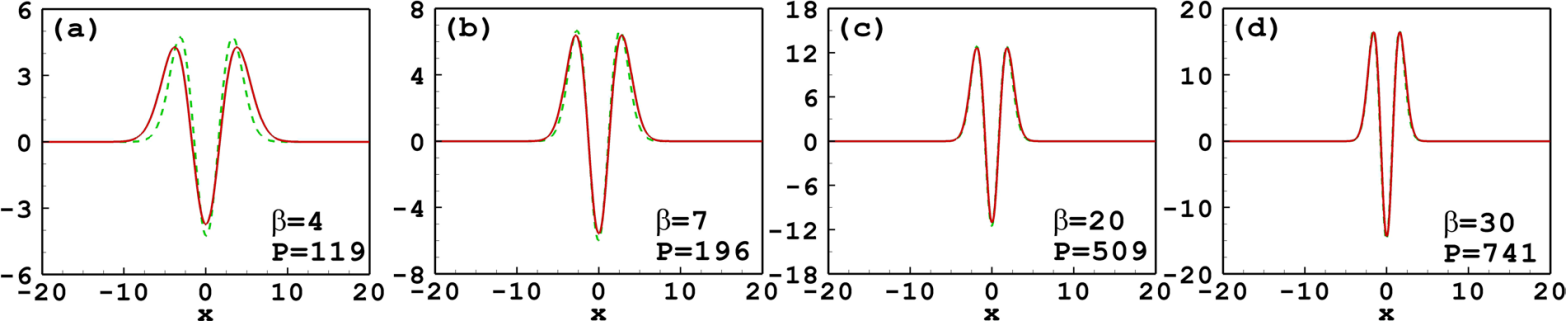
Profiles of the tripole-mode soliton with different soliton powers and propagation constants. The solid line and the dashed line denote, respectively, the approximate solutions and the numerical ones. Parameter *w*_*m*_ = 10 for all cases.

Figure 3 shows the profiles of the tripole-mode soliton with different soliton powers. It is found that the approximate solutions agree well with the numerical solutions for *β* = 30 and 20. When *β* decreases to 7, the approximate solution becomes a little inaccurate, and when *β* decreases to 4, it becomes even worse. The degrees of nonlocality are 3.19, 2.78, 1.93, and 1.59 for *β* = 30, 20, 7, and 4, respectively. It is found that when the nonlocality degree is 1.59, i.e. *β* = 4, an obvious deviation appears between the variational solution and the numerical one. The reason is that the Taylor series truncation (18) is starting to break down for this low *w*_*m*_. So we can conclude that if the response fuction is expanded to the second order, the approximate solutions are valid only for the strongly nonlocal case.

### Quadrupole-mode soliton solutions

For the quadrupole-mode solitons in (1 + 1)-dimensional NLCs, we take the following ansatz solution

25 $$q(x,z)=a(\frac{2{x}^{3}}{{w}^{2}}-3x)\exp (-\frac{{x}^{2}}{2{w}^{2}}){e}^{i\theta (z)}.$$

According to Eq. (17), one can easily get the width of quadrupole-mode solitons is ${w}_{R}=\sqrt{14}w$. Based on Eqs (13), (15), (18) and (25), the expression of $[ {\mathcal L} ]$ is obtained as follows

26 $$\begin{matrix}[ {\mathcal L} ] & = & -\frac{21}{4}\sqrt{\pi }{a}^{2}w+\frac{9{a}^{4}{w}^{6}[-687\sqrt{2\pi }w{w}_{m}+128\pi (7{w}^{2}+2{w}_{m}^{2})]}{1024{w}_{m}^{3}}\\  &  & -3{a}^{2}\sqrt{\pi }{w}^{3}\theta ^{\prime} (z).\end{matrix}$$

Following the same process in the previous section, one can obtian

27 $$a=\frac{16\sqrt[4]{2}\sqrt{7{w}_{m}^{3}}}{\sqrt{3{w}^{6}(687{w}_{m}-896\sqrt{2\pi }w)}},$$

28 $$\beta =-\frac{7(2688\sqrt{2\pi }{w}^{2}-3435w{w}_{m}+512\sqrt{2\pi }{w}_{m}^{2})}{4{w}^{3}(896\sqrt{2\pi }w-687{w}_{m})},$$

and the soliton power

29 $$P=\frac{1792\sqrt{2\pi }{w}_{m}^{3}}{(687{w}_{m}-896\sqrt{2\pi }w){w}^{3}}.$$

Similar to Eqs (22–24) and (27–29) are valid only when $687{w}_{m}-896\sqrt{2\pi }w > 0$. Comparing with the SMM which is valid for the strongly nonlocal case, it is found that the soliton powers are different. The soliton powers for tripole-mode and quadrupole-mode solitons in (1 + 1)-dimensional NLCs are not equal, and it is larger for quadrupole-mode solitons than tripole-mode solitons with the same parameters. However the multipole solitons and the higher-order solitons are all the same in SMM^12–16^. Furthermore, for the the strongly nonlocal limit (i.e. ${w}_{m}\gg w$), the soliton powers in (1 + 1)-dimensional NLCs are both inversely proportional to *w* ^3^ approximately, while it is inversely proportional to *w* ^4^ in SMM.

**Figure 4:**
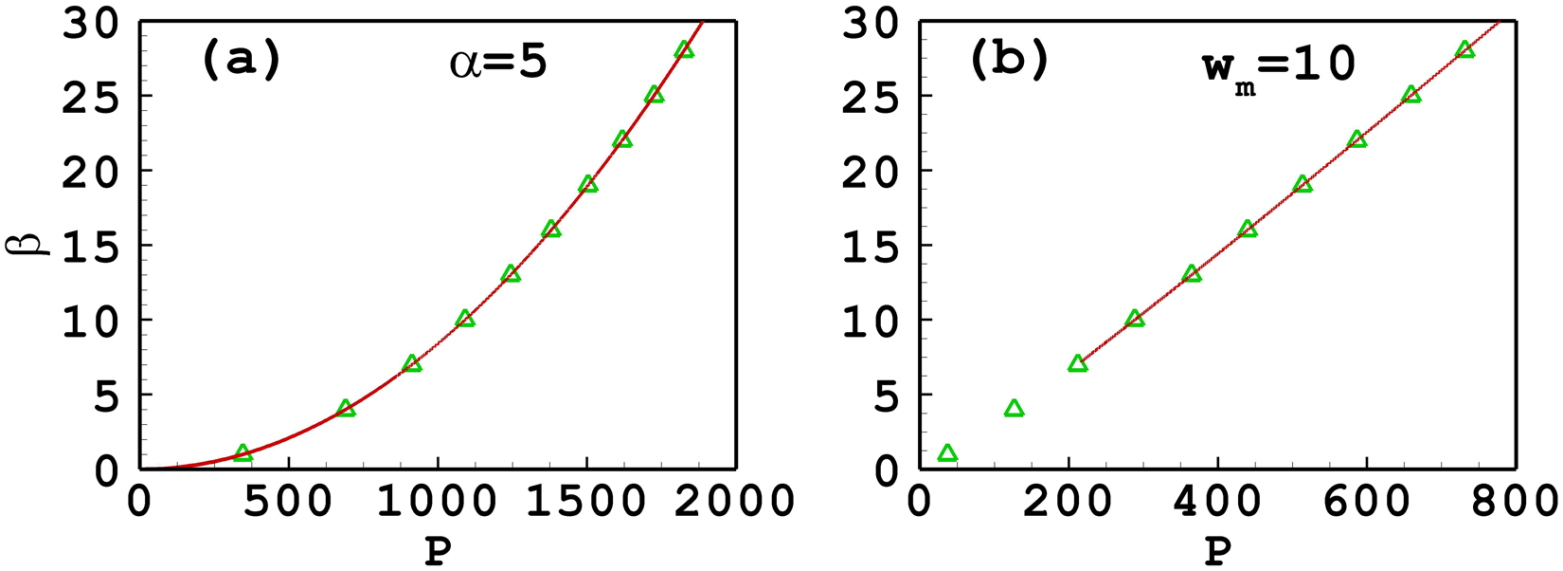
The propagation constant versus the power of quadrupole-mode solitons. (**a**) The degree of nonlocality is fixed at *α* = 5; (**b**) the width of the response function is fixed at *w*_*m*_ = 10. The solid line and the triangles denote, respectively, the approximate solutions and the numerical ones.

**Figure 5:**
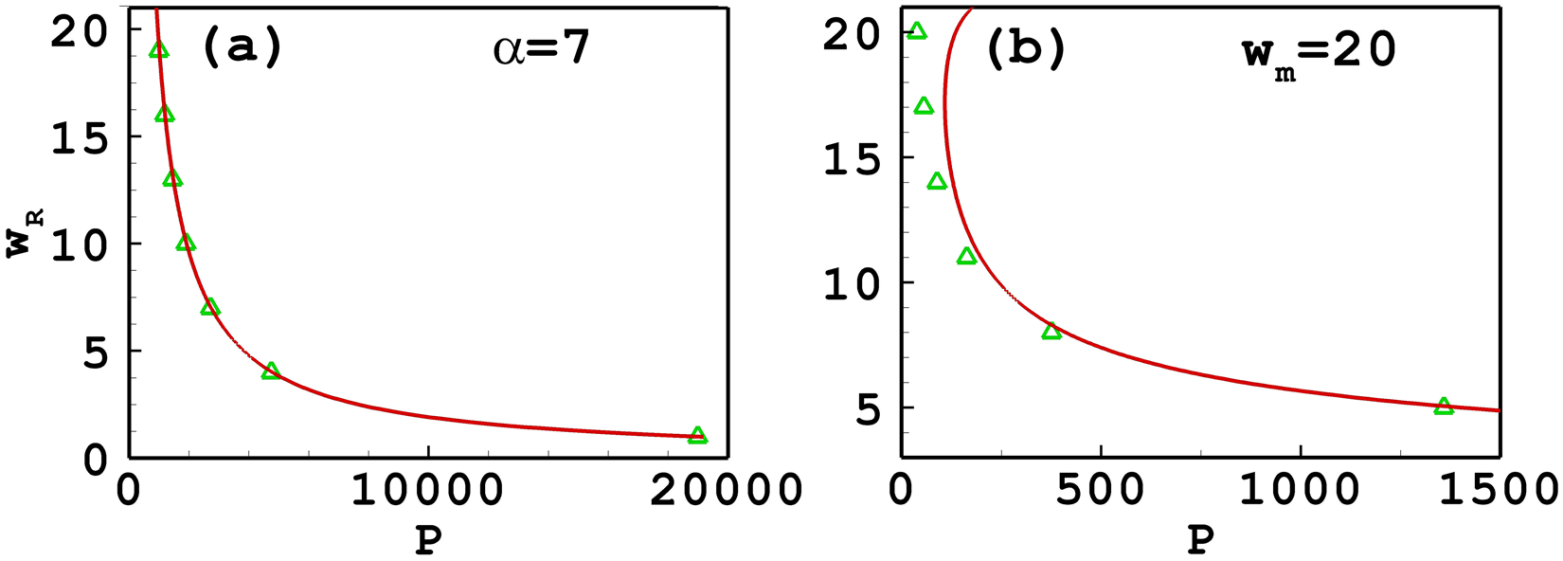
The soliton width versus the power of quadrupole-mode solitons. (**a**) The degree of nonlocality is fixed at *α* = 7; (**b**) the width of the response function is fixed at *w*_*m*_ = 20. The solid line and the triangles denote, respectively, approximate solutions and the numerical ones.

**Figure 6:**
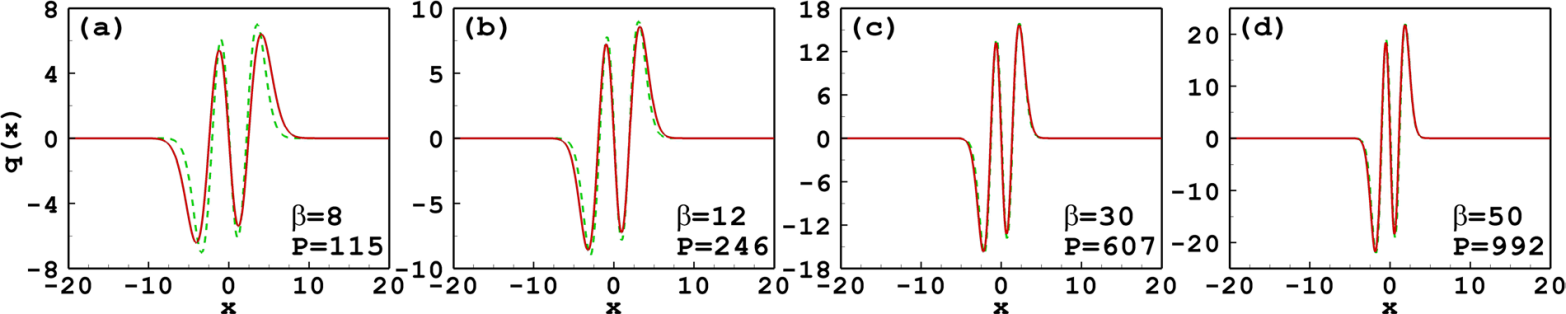
Profiles of the quadrupole-mode soliton with different soliton powers and propagation constants. The solid line and the dashed line denote, respectively, the approximate solutions and the numerical ones. Parameter *w*_*m*_ = 10 for all cases.

Figures 4 and 5 illustrate the propagation constant and the soliton width versus the power of quadrupole-mode solitons, which are similar to the tripole-mode solitons. When the degree of nonlocality is fixed at 5 or 7, the approximate results are in good agreement with the numerical ones [see Figs 4(a) and 5(a)]. When *w*_*m*_ is fixed, the soliton width increases with the decrease of the soliton power [see Fig. 5(b)], which indicates the nonlocality degree decreases. As a results, the approximate result becomes invalid gradually. Especially when *β* < 7.17, approximate results do not exist any more [see Fig. 4(b)]. Figure 4 shows that the slope of the power versus propagation constant is positive, similar to the case of tripole solitons, which implies a stable propagation of solitons. Figure 6 presents the profiles of the quadrupole-mode soliton with different soliton powers and propagation constants. It is found that the approximate solutions agree well with the numerical solutions for *β* = 50 and 30. When *β* decreases to 12, the approximate solution has a little inaccuracy, and when *β* decreases to 8, it becomes even worse. We also calculate the nonlocal parameter *α*, and find that it is equal to 3.02, 2.53, 1.85, and 1.61 for *β* = 50, 30, 12, and 8, respectively.

### More accurate approximate solutions

In the above sections, the response function is only expanded to the second order. So with the degree of nonlocality decreasing, the approximate results become invalid gradually. In order to get a more exact approximate solution, one can expand the response function to the higher orders. The higher orders the response function is expanded into, the more exact the approximate solutions are. Of course, the calculations are more complicated. As an example, we expand *R*(*x*) to the fourth order, i.e.,

30 $$R(x)\simeq \frac{1}{2{w}_{m}}(1-\frac{|x|}{{w}_{m}}+\frac{{x}^{2}}{2{w}_{m}^{2}}-\frac{{|x|}^{3}}{6{w}_{m}^{3}}+\frac{{x}^{4}}{24{w}_{m}^{4}}).$$

Following the same process in the above section, one can obtain the approximate solutions of tripole-mode solitons

31 $$a=\frac{4\sqrt[4]{8}\sqrt{5{w}_{m}^{5}}}{\sqrt{-304\sqrt{2\pi }{w}^{7}+649{w}^{6}{w}_{m}-160\sqrt{2\pi }{w}^{5}{w}_{m}^{2}+145{w}^{4}{w}_{m}^{3}}},$$

32 $$\beta =\frac{5}{228}(-\frac{114}{{w}^{2}}-\frac{30{w}_{m}^{2}}{{w}^{4}}-\frac{12331{w}^{4}{w}_{m}+5325{w}^{2}{w}_{m}^{3}-2496\sqrt{2\pi }w{w}_{m}^{4}-4350{w}_{m}^{5}}{-304\sqrt{2\pi }{w}^{7}+649{w}^{6}{w}_{m}-160\sqrt{2\pi }{w}^{5}{w}_{m}^{2}+145{w}^{4}{w}_{m}^{3}}),$$

and the soliton power

33 $$P=\frac{320\sqrt{2\pi }{w}_{m}^{5}}{(-304\sqrt{2\pi }+649{w}^{2}{w}_{m}+160\sqrt{2\pi }w{w}_{m}^{2}-145{w}_{m}^{3}){w}^{3}}.$$

**Figure 7:**
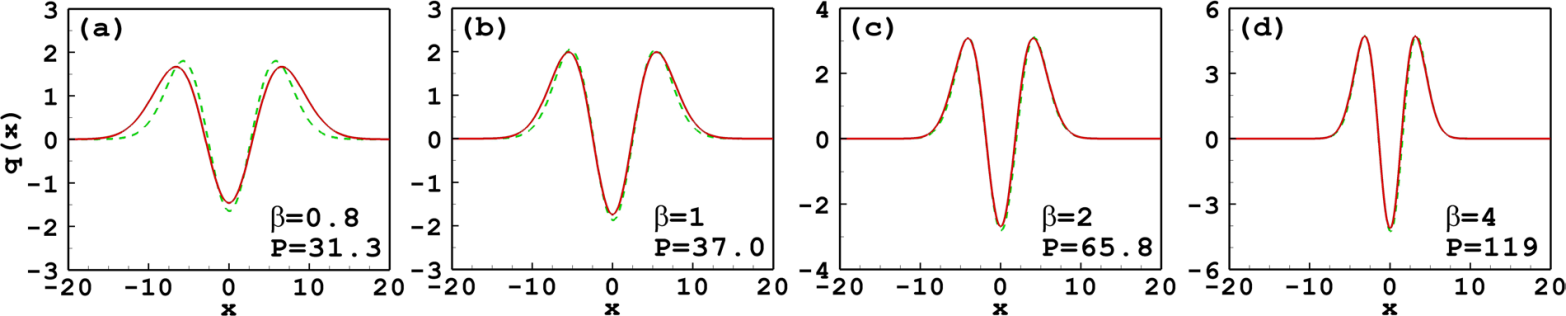
Profiles of the tripole-mode soliton with different soliton powers and propagation constants by expanding the response function to the fourth order. The solid line and the dashed line denote, respectively, the approximate solutions and the numerical ones. Parameter *w*_*m*_ = 10 for all cases.

Similar to Eqs (22–24) and (31–33) are valid only when $-304\sqrt{2\pi }{w}^{7}+649{w}^{6}{w}_{m}-160\sqrt{2\pi }{w}^{5}{w}_{m}^{2}+145{w}^{4}{w}_{m}^{3} > 0$. In the previous section, it is found that the approximate tripole-mode solution is inaccurate when *β* = 4, and even it is not obtained when *β* < 3.68. In order to show the improvement of the approximate solutions obtained by expanding the respond function to the fourth order, Fig. 7 shows the comparison between the approximate results and the numerical ones. It is found that the approximate solutions are in good agreement with the numerical ones when *β* = 4 and even *β* = 2. For the case of *β* = 2, the degree of nonlocality is about 1.23, which already belongs to the general nonlocality. When *β* = 1, the approximate solution has a little inaccuracy, and when *β* = 0.8, it becomes even worse. The degrees of nonlocality for *β* = 1 and *β* = 0.8 are, respectively, 0.91 and 0.76.

Furthermore, if *R*(*x*) is expanded to the tenth order, i.e.,

34 $$\begin{matrix}R(x) & \simeq  & \frac{1}{2{w}_{m}}(1-\frac{|x|}{{w}_{m}}+\frac{{x}^{2}}{2{w}_{m}^{2}}-\frac{1}{\mathrm{3!}}\frac{{|x|}^{3}}{{w}_{m}^{3}}+\frac{1}{\mathrm{4!}}\frac{{|x|}^{4}}{{w}_{m}^{4}}-\frac{1}{\mathrm{5!}}\frac{{|x|}^{5}}{{w}_{m}^{5}}+\frac{1}{\mathrm{6!}}\frac{{|x|}^{6}}{{w}_{m}^{6}}-\frac{1}{\mathrm{7!}}\frac{{|x|}^{7}}{{w}_{m}^{7}}\\  &  & +\frac{1}{\mathrm{8!}}\frac{{|x|}^{8}}{{w}_{m}^{8}}-\frac{1}{\mathrm{9!}}\frac{{|x|}^{9}}{{w}_{m}^{9}}+\frac{1}{\mathrm{10!}}\frac{{|x|}^{10}}{{w}_{m}^{10}}),\end{matrix}$$

similarly, one can get the approximate solutions of tripole-mode solitons,

35 $$a=\frac{40{w}_{m}^{5}}{{w}^{2}}\sqrt{\frac{42{w}_{m}}{(A+B)}},$$

36 $$\beta =\frac{\mathrm{5(}C+D)}{36{w}^{3}(A+B)},$$

and

37 $$P=\frac{134400\sqrt{\pi }{w}_{m}^{11}}{{w}^{3}(A+B)},$$

where

$$\begin{matrix}A & = & 2\sqrt{2}{w}_{m}(12241{w}^{8}+38815{w}^{6}{w}_{m}^{2}+74515{w}^{4}{w}_{m}^{4}+68145{w}^{2}{w}_{m}^{6}+15225{w}_{m}^{8}),\\ B & = & -35\sqrt{\pi }(271{w}^{9}+1048{w}^{7}{w}_{m}^{2}+2640{w}^{5}{w}_{m}^{4}+3648{w}^{3}{w}_{m}^{6}+1920w{w}_{m}^{8}),\\ C & = & -2\sqrt{2\pi }w{w}_{m}(159133{w}^{8}+548955{w}^{6}{w}_{m}^{2}+1207143{w}^{4}{w}_{m}^{4}\\  &  & +1431045{w}^{2}{w}_{m}^{6}+685125{w}_{m}^{8}),\\ D & = & 63\pi (1897{w}^{10}+7860{w}^{8}{w}_{m}^{2}+22000{w}^{6}{w}_{m}^{4}+36480{w}^{4}{w}_{m}^{6}\\  &  & +28800{w}^{2}{w}_{m}^{8}+7680{w}_{m}^{10}).\end{matrix}$$

It should be noted that Eqs (35–37) are valid only when *A* + *B* > 0.

**Figure 8:**
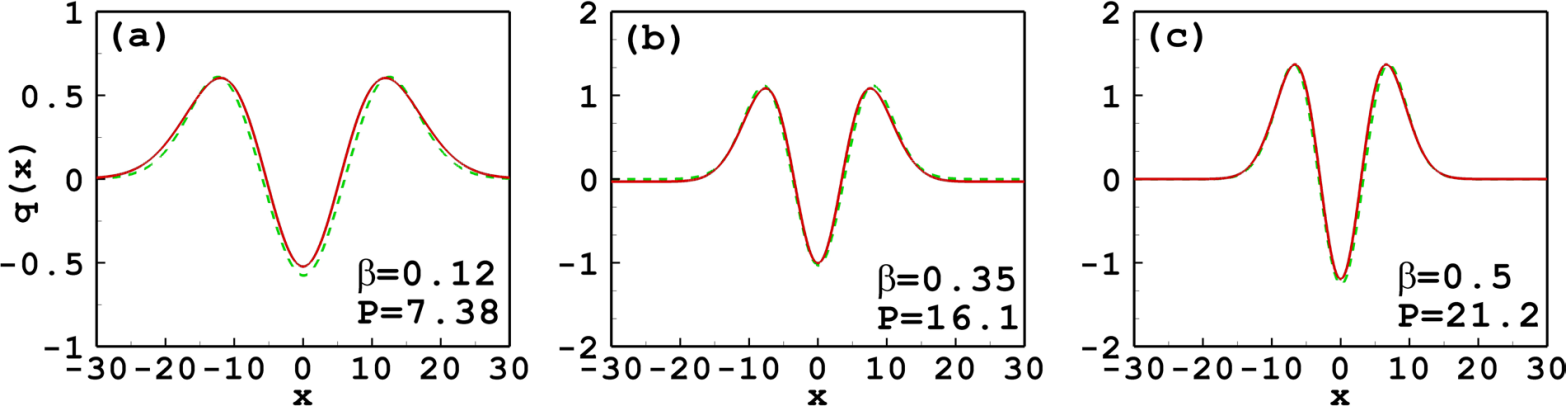
Profiles of the tripole-mode soliton with different soliton powers and propagation constants by expanding the response function to the tenth order. The solid line and the dashed line denote, respectively, the approximate solutions and the numerical ones. Parameter *w*_*m*_ = 10 for all cases.

Figure 8 shows the profiles of the tripole-mode soliton obtained by expanding the response function to the tenth order. When *β* = 0.5, 0.35 and 0.12, the corresponding degrees of nonlocality are, respectively, 0.753, 0.657 and 0.417, which all belong to the case of the general nonlocality. Therefore, the approximate solutions can be improved by expanding the response function to the higher orders.

**Figure 9:**
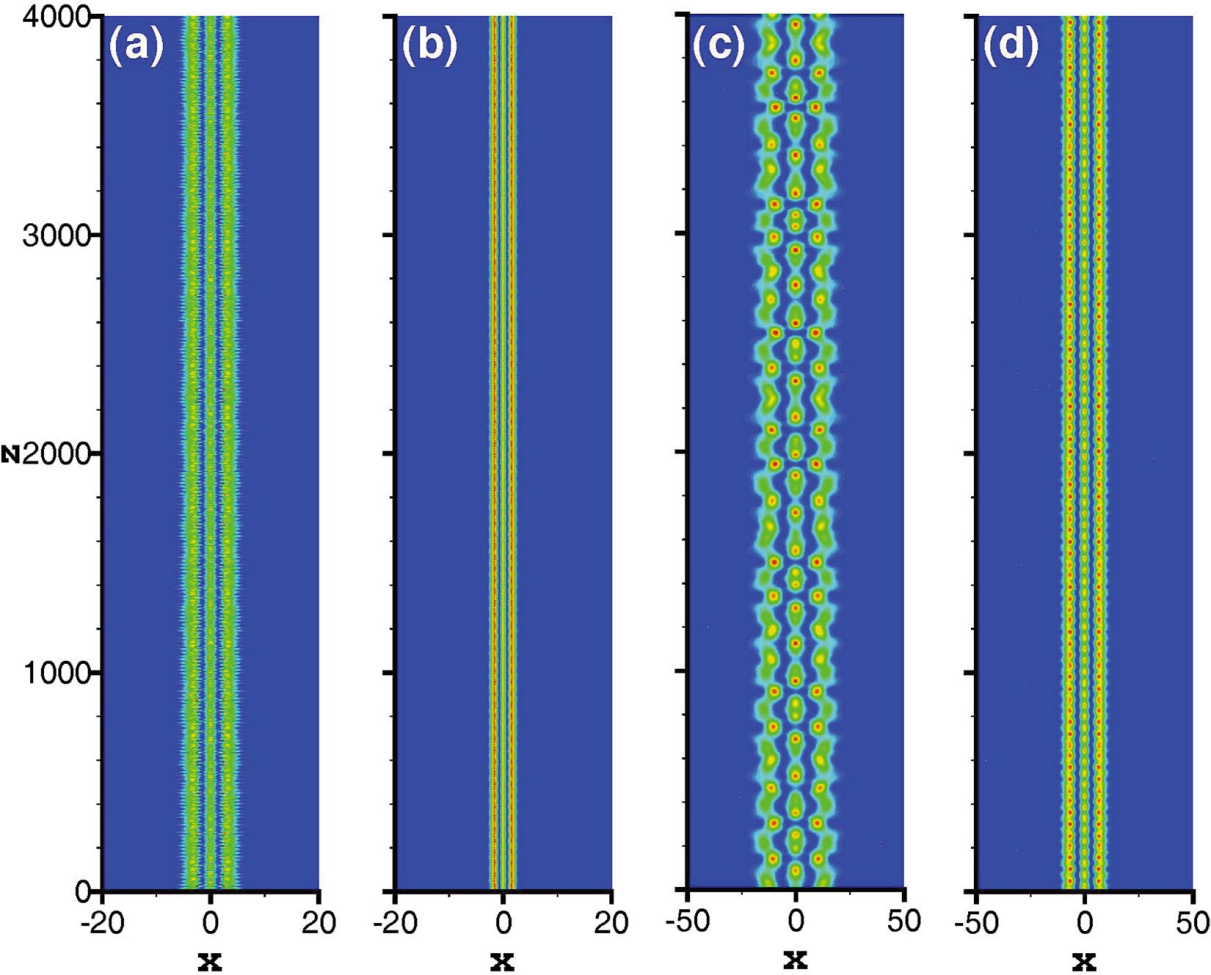
Propagation of tripole-mode solitons in the presence of 1% white input noise. The profiles of Fig. 3(a,d) are employed as the input shapes of solitons in (**a**,**b**), and the profiles of Fig. 8(a,c) are employed as the input shapes of solitons in (**c**,**d**).

**Figure 10:**
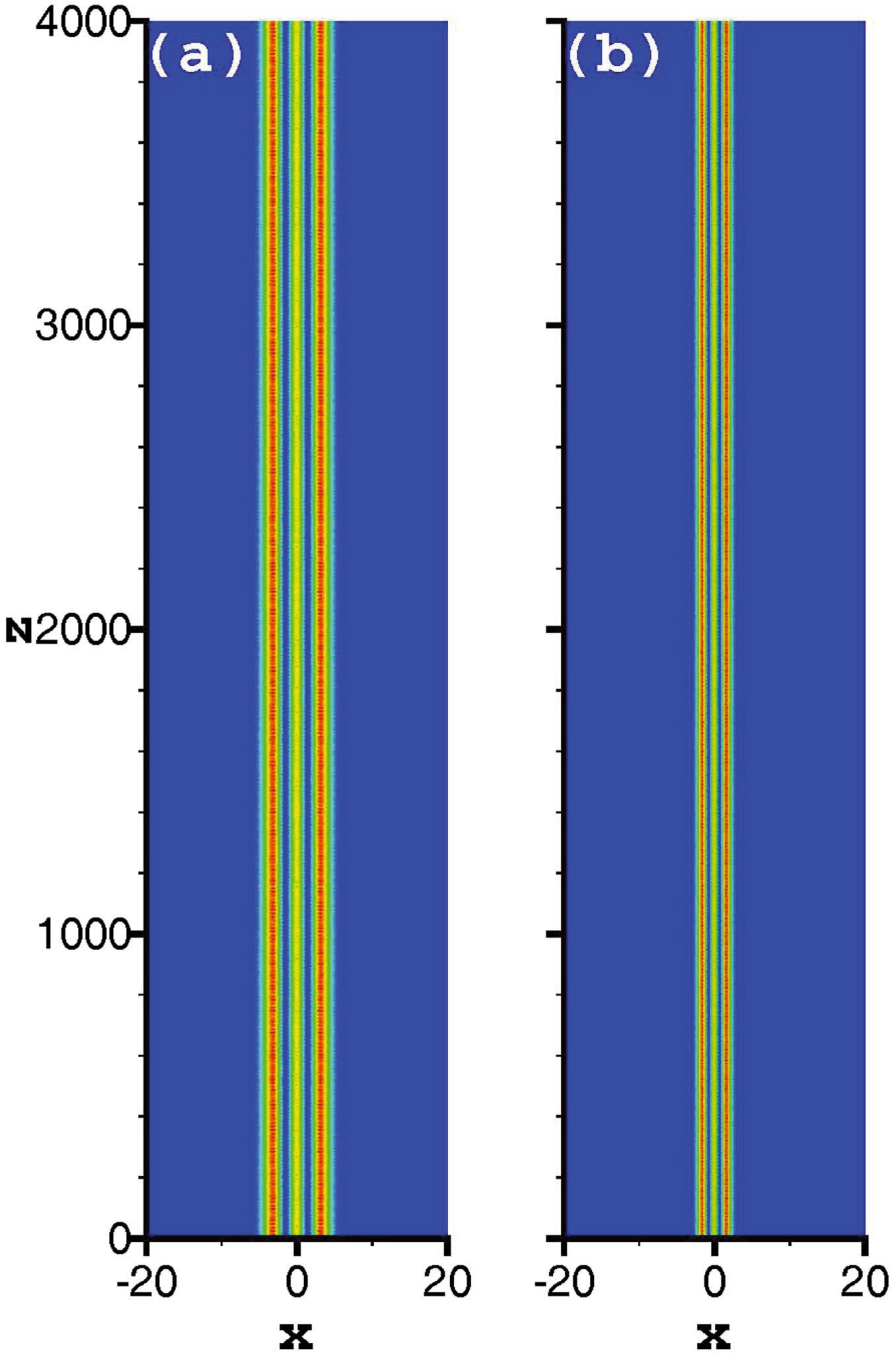
Propagation of tripole-mode solitons in the presence of 1% white input noise by expanding the response function to the tenth order. (**a**,**b**) Correspond to the cases of Fig. 3(a,d), respectively, and the input shapes in (**a**,**b**) are obtained based on Eqs (35–37).

**Figure 11:**
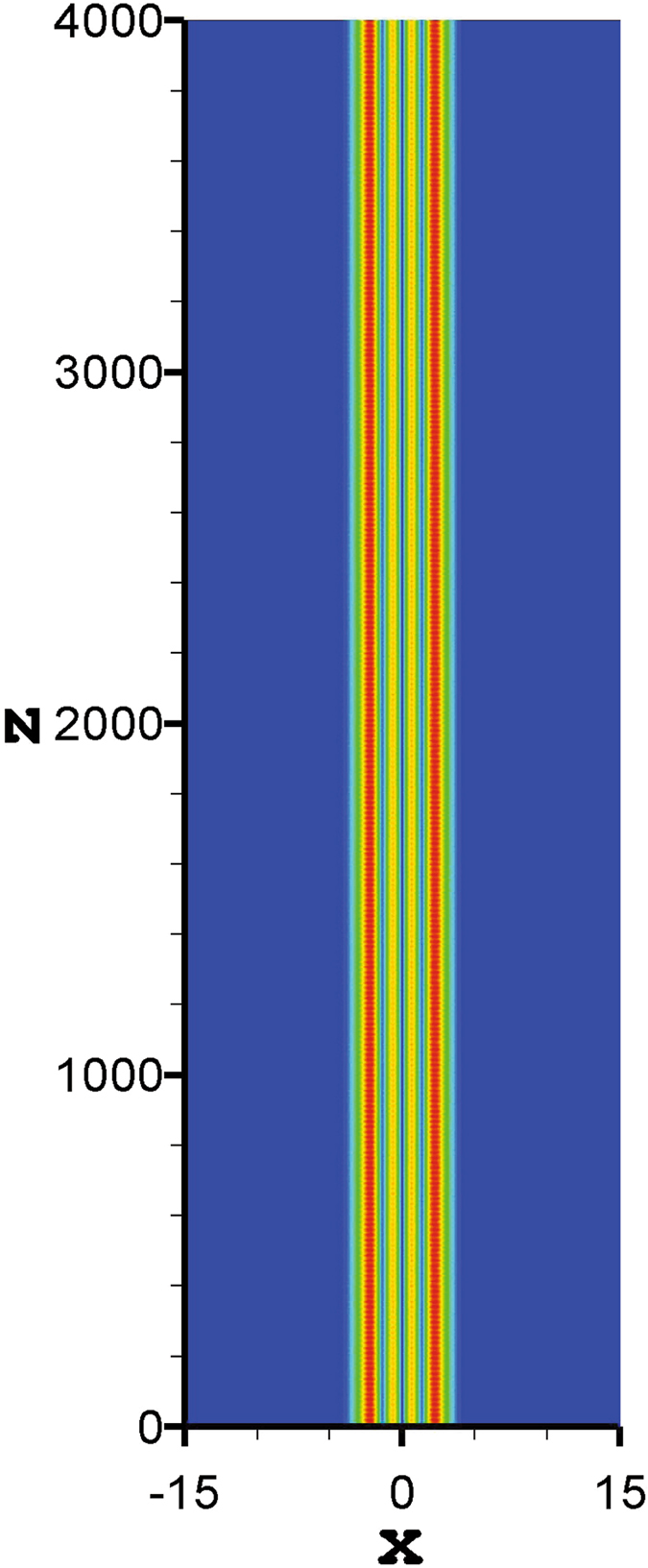
Propagation of quadrupole-mode solitons in the presence of 1% white input noise. The parameters are taken from Fig. 6(c).

Although it has been proven that in a nonlinear medium with an exponential-decay nonlocal response, the soliton bound states are stable if the solitons contain fewer than five-poles^21^. In order to confirm the validity of our results, we take the tripole-mode soliton as an example and simulate the propagation based on Eqs (8) and (9) directly. Here we take the split-step Fourier method^54^ to simulate the soliton propagation. As expected, for the strongly nonlocal case, the approximate results obtained by expanding the response function to the second order are accurate, and the solitons can stably propagate for a long distance [see Fig. 9(b)]. With the decrease of the nonlocality degree, the approximate results become inaccurate gradually, and irregular oscillations occur during propagation [see Fig. 9(a)]. However, if the nonlocal response function is expanded to the tenth order, the accuracy and the validity range of the approximate solutions increase. The approximate soliton can still keep a relatively stable propagation [see Fig. 9(d)], but it can not even be obtained if the response function is only expanded to the second order. In Fig. 9(c), because the degree of nonlocality is weaker than that in Fig. 9(d), the irregular oscillations appear more obviously. The oscillations of Fig. 9(c,d) are just typical behaviour for NLS-type equations. For such equations an initial condition near a solitary wave will evolve to the solitary wave with the amplitude and width displaying decaying oscillations. All these oscillations shows the degree of accuracy of the variational solutions. Therefore, we can conclude that by expanding the respond function to the higher orders, the accuracy of the approximate soliton solutions is improved. Corresponding to the cases of Figs 9(a,b) and 10 illustrates the propagation of the tripole-mode solitons by expanding the response function to the tenth order, which shows a stable propagation of solitons. Especially, when *β* = 4, the approximate soliton obtained by expanding the response function to the tenth order is still valid [see Fig. 10(a)]. Contrarily, it is already invalid when the response function is expanded to the second order. As another example, Fig. 11 illustrates the stable propagation of a quadrupole-mode soliton, which confirms the validity of the approximate variational quadrupole-mode soliton solutions.

At last, for the completeness, we also give the solutions of quadrupole-mode solitons as follows when the response function is expanded to the tenth order.

38 $$a=\frac{16{w}_{m}^{5}\sqrt[4]{2}\sqrt{35{w}_{m}}}{{w}^{3}\sqrt{F}},$$

39 $$\beta =-\frac{G}{36{w}^{3}F},$$

and

40 $$P=\frac{26880\sqrt{2\pi }{w}_{m}^{11}}{{w}^{3}F},$$

where

$$\begin{matrix}F & = & -6405\sqrt{2\pi }{w}^{9}+28921{w}^{8}{w}_{m}-18840\sqrt{2\pi }{w}^{7}{w}_{m}^{2}\\  &  & +68947{w}^{6}{w}_{m}^{3}-35280\sqrt{2\pi }{w}^{5}{w}_{m}^{4}\\  &  & +97355{w}^{4}{w}_{m}^{5}-35520\sqrt{2\pi }{w}^{3}{w}_{m}^{6}+64365{w}^{2}{w}_{m}^{7}\\  &  & -13440\sqrt{2\pi }w{w}_{m}^{8}+10305{w}_{m}^{9},\\ G & = & -564921\sqrt{2\pi }{w}^{10}+2631811{w}^{9}{w}_{m}\\  &  & -1780380\sqrt{2\pi }{w}^{8}{w}_{m}^{2}+6825753{w}^{7}{w}_{m}^{3}\\  &  & -3704400\sqrt{2\pi }{w}^{6}{w}_{m}^{4}+11040057{w}^{5}{w}_{m}^{5}-4475520\sqrt{2\pi }{w}^{4}{w}_{m}^{6}\\  &  & +9461655{w}^{3}{w}_{m}^{7}-2540160\sqrt{2\pi }{w}^{2}{w}_{m}^{8}\\  &  & +3246075w{w}_{m}^{9}-483840\sqrt{2\pi }{w}_{m}^{10}.\end{matrix}$$

Note that Eqs (38–40) are valid only when *F* > 0.

## Discussion

By applying the variational approach, we obtain the approximate analytical expressions of tripole-mode and quadrupole-mode solitons in nonlinear media with an exponential-decay nonlocal response. It is found that with the same parameters, the soliton power of the quadrupole-mode solitons is larger than that of the tripole-mode solitons, which is much differnt from the SMM (In SMM, the soliton powers with different multipoles are the same^12, 15^). The numerical simulations are carried out to illustrate the accuracy of the approximate solutions. The results show that the accuracy of the approximate solutions is only related with the degree of nonlocality. For the strongly nonlocal case, if the response function is expanded to the second order, the approximate soliton solutions are in good agreement with the numerical ones. With the degree of nonlocality decreasing, the approximate solutions become invalid gradually. Furthermore, by expanding the respond function to the higher orders, one can improve the accuracy of the approximate solutions. The higher orders the response function is expanded to, the more exact the approximate solutions are. If the response function is expanded to the tenth order, the approximate solutions are still valid for the general nonlocal case. Since a surface soliton in nonlocal nonlinear media can be regarded as a half of a bulk soliton with an antisymmetric amplitude distribution^43, 44^, the results on quadrupole-mode solitons here may also be helpful for the investigation of the surface dipole nonlocal solitons.
